# Healthcare Simulation: An effective way of learning in health care

**DOI:** 10.12669/pjms.39.4.7145

**Published:** 2023

**Authors:** Munazza Saleem, Zuhera Khan

**Affiliations:** 1 Dr. Munazza Saleem, M.B.B.S., MHST, Liaquat University of Medical and Health Sciences, Jamshoro, Hyderabad, Pakistan. Athabasca University, Faculty of Health Disciplines, Calgary, Alberta, Canada.; 2 Dr. Zuhera khan, FCPS (Plast) Assistant Professor, Plastic Surgery, Indus Medical College, Tando Muhammad Khan, Pakistan

**Keywords:** Simulation, Healthcare teaching, Manikins, Simulation in health care, Pedagogy, simulation-based learning, Debriefing, Educational strategies, Use of simulation in health care

## Abstract

**Background and Objective:**

Simulation-based learning has been a part of teaching in healthcare for a long time; however, in recent decades, simulation-based learning has been adopted by a significant number of healthcare institutes at different levels to improve practical skills, confidence, and preparedness to ensure patient safety and its application in real-life situations towards better patient care. The main objective of this paper was to use existing literature to explore aspects of simulation in healthcare teaching.

**Methods:**

It is a narrative review on simulation in healthcare that was conducted by using various search engines for English-language articles published between 2010 and August 2020. The main search terms were simulation, healthcare teaching, and simulation in healthcare. All articles found relevant to the title and/or abstract were retrieved. Searches were conducted using the academic databases PubMed, Google Scholar, MEDLINE, CINAHL, and Athabasca University (AU) library site. The studies were reviewed if they were considered relevant to the search by the primary authors.

**Results:**

Thirty-nine articles, which met the pre-set criteria, were analyzed and employed as a reference in this paper to support the idea that simulation is an effective way of learning in healthcare.

**Conclusion:**

This paper reviewed various aspects of simulation, including its background, philosophies, and highlighted the advantages and disadvantages of incorporating simulation as a pedagogical approach into current educational curriculums for healthcare students. Furthermore, it presents a brief discussion on the current uses of simulation, followed by the educational strategies related to simulation and the importance of debriefing in simulation activities.

## INTRODUCTION

Simulation-based learning is one of the main components of health care education, which has proliferated tremendously in the last few decades and is widely accepted by the teaching community as a method to expedite skill training and assessment.^1^ Simulation-based education encompasses the knowledge, skills, and behaviors attained by healthcare professionals toward better patient-centric care.^2^ Due to legal and ethical rights, the traditional ways of learning without consent from animals, cadavers, and patients are becoming less acceptable.^3^ Simulation grants an opportunity to recreate the rare scenarios for learning that are not seen in routine hospital settings; it not only contributes to individual learning but can be applied to teams and organizations.^4^ Today, a number of health care institutes around the world have adopted simulation as a learning tool for skills acquisition and decision-making through real-life situational experiences without compromising patient safety.^5^ The embodiment of simulation in learning has been intended to replicate the real world and will provide novice healthcare trainees with the opportunity to practice procedures and diagnostic methods on computer-guided models in a professional manner.^6^ Simulation-based learning has prompted healthcare professionals to work inter-professionally towards collaborative patient-focused care.^7^,^8^

Considering the significance of simulation in healthcare teaching, the main objective of this paper is to use existing literature to explore aspects of simulation in healthcare teaching. This narrative study is intended to review literature and synthesize the findings of literature retrieved from searches of computerized databases, hand searches and authoritative texts to gather the relevant attributes to support the notion that simulation is an effective way of learning in healthcare. The paper is organized to discuss the background and highlight the advantages and disadvantages of incorporating simulation as a pedagogical approach into current educational curriculums for healthcare students; furthermore, it presents a brief discussion on the current uses of simulation, followed by the educational strategies related to simulation and the importance of debriefing in the simulation activities. Finally, as to why future research is needed to increase our current knowledge regarding simulation-based learning in healthcare is debated.

## METHODS

A literature review was conducted to identify referenced content and gather relevant papers as the knowledge foundation to formulate this paper. Searches were conducted using the academic databases PubMed, Google Scholar, MEDLINE, CINAHL, and Athabasca University (AU) library site. Keywords and various combinations of key terms were utilized to search the potential eligible studies included “simulation”, “healthcare teaching”, “manikins”, “simulation in health care”, “pedagogy”, “simulation-based learning”, “debriefing”, “educational strategies” and “use of simulation in health care”. The search strategy yielded hundreds of potential articles, which were further narrowed by reading the titles and abstracts of the articles. The articles were hand-selected based on the relevance of the topic to conducting this literature review by a primary researcher.

Following sifting, thirty-nine articles that met the pre-set criteria were included in the paper. The criteria used to screen all the articles included: (a) Studies published within ten years (2010–2020), which allowed for the most up-to-date knowledge on the topic of interest, (b) Studies published in English; (c) Articles focused on the teaching and learning practices in simulation in health care; and (d) only peer-reviewed articles that are available in full text. All the additional retrieved articles that were case reports, letters to the editor, or studies older than ten years were excluded at the screening step. All the articles published in a language other than English were eliminated, despite meeting the pre-set criteria.

### Historical background of simulation:

Improving knowledge and skill development has been a crucial component of the learning process across a variety of simulations, from flight simulators to healthcare simulators. Simulation in its preliminary stages has been practiced for decades in the form of physical models of anatomical parts of the human body.^4^ Simulation-based teaching in health care was first introduced in the 9th century by a French midwife, Madame du Coudray, to better train and illustrate childbirth-related complications to midwives.^4^ Simulators in health care education have a long history. As science, technology, and teaching techniques have improved, simulation has evolved to be exceptional and innovative learning and teaching approach.^4^

Simulation is derived from the Latin word “simulare” which means “to copy”. Simulation is defined as “the imitation of some real thing, state of affairs, or process”.^4^ Recently, simulation has been divided into five categories: (I) Electronic patients (II) Computer patient (III) Part task trainers (IV) Standardized patients (SPs) (V) Verbal.^9^ The educational strategy to achieve specific goals related to learning or evaluation in a safe and supportive environment is referred to as simulation-based learning.^4^

### Examples of simulation:

Manikins are duplicate models of the human body or parts of the human body, adopted for the training of clinical skills of students. A manikin can be classified by its level of realism, from a low-fidelity manikin to a high-fidelity manikin.^10^

### Low-fidelity:

Low-fidelity manikins are segmented clinical task trainers usually used for basic skills training. They are static and competent in performing specific and restricted tasks that lack realism and situational context.^10^ An intravenous (IV) arm for the administration of injections, cardio-pulmonary resuscitation (CPR) for resuscitation, and a pig foot for wound drainage are examples of low-fidelity manikins.^10^

### High- fidelity:

High-fidelity manikins are full-body simulated patients incorporated with advanced computer technology and control. They give more resemblance to real patients through the demonstration of vital signs such as pupil dilation, the rate of pulse and breath through chest rise. High-fidelity manikins are used in learning a variety of high-stakes situations.^10^,^11^ Good examples of a high-fidelity simulator are the model-driven METI Human Patient Simulator (HPS)^11^ and the NOELLE simulator, which is an instructor-driven obstetric simulator for postpartum hemorrhage.^10^

### Theories and philosophies linking to simulation:

Simulation-based education is a student-centered approach grounded in learning theories based on collaboration and constructivism.^12^ The simulation creates an environment that places tremendous importance on active and collaborative learning for students, allowing them to build new ideas or transform old concepts based on their current and prior knowledge, resulting in long-lasting learning.^13^ Simulation learning is generally conducted in a group to assume the different roles and perspectives of a healthcare professional called a “constructive group”.^14^ The most common theory adopted to guide and develop simulation-based education is Kolb’s Experiential Learning Theory (ELT) (Kolb, 1974). The framework of Kolb’s ELT theory comprises encountering a new experience, reflecting on the experience, analyzing and developing the conclusions, and active experimentation, which empowers learners to apply theory competently and safely in real-life settings.^15^

The literature strongly indicates that simulation-based learning is congruent with the behaviorist philosophy since it focuses on repetition and allows students to refine their skills.^13^,^16^ The implementation of learning by simulation involves rote learning, repetition of skills, pre-briefing, de-briefing, and modular learning. These are all predominant behaviorist approaches to creating the foundation knowledge and skills of a healthcare student.^14^

### Effectiveness of simulation based education:

Top of Form Simulation-based education is an exceptional educational approach that provides appropriate learning opportunities to students in understanding complex concepts;^17^ moreover, it allows mastery in clinical judgement, decision-making abilities, and enhances critical thinking.^18^-^20^ Besides these non-technical skills, like communication, leadership, and teamwork, they can be learned through simulation.^21^ Research studies have also shown that, apart from individual and team learning, simulation tools provide an opportunity to test administrative performance and problem-solving skills.^5^ Numerous scenarios can be recreated, including some of the rare conditions that enhance the ability of students to improve learning and augment patient safety. Thus, simulation is an opportunity for immersive and experiential learning with the acquisition of theoretical knowledge and assessment skills for the care of the patient.^17^

Several research investigations have proven that simulation-based learning has improved the healthcare system as a whole and not just on an individual basis.^1^,^11^ A quasi-experimental study was carried out at the Neonatal Intensive Care Unit of Marmara University in Istanbul, Turkey. The study aimed to evaluate the effects of simulation education on newborn evaluation and care skills in fourth-year midwifery students who selected the Intern Newborn course in the 2017–2018 fall and spring semesters. The study aimed to bridge the knowledge gaps and enhance clinical judgement and patient outcomes among the control and experimental groups. The “Delivery Room Neonatal Initial Evaluation/Care” subject was instructed via a computer-aided simulation model under the Newborn Evaluation Guide in the experimental group of students. After statistical analyses of the students from both groups, it was determined that those who had received simulation-based training (experimental group) were more competent than the control group.^22^ Another study was found in support of simulation, which was conducted in the emergency department to determine the impact of implementing scenarios’ simulation on idle and service time.

The study included patients who received services in the emergency department (ED). The findings revealed a considerable decline in service times, which means faster service is provided to customers after simulation implementation.^23^ A randomized study was conducted by Keleekai and colleagues to improve clinical skills and knowledge between two groups of nurses for the insertion of a peripheral intravenous catheter. The intervention group, which received a two-hour training, revealed an improvement in knowledge and skills, as compared to the wait-list group.^24^

### Advantages of simulation based education:

The large body of evidence predominately recommends simulation as an effective teaching strategy that is beneficial both for the learner and the educator.^1^ Simulation in teaching is vast and growing at a rapid rate as an educational tool to facilitate self-reliance, problem-solving abilities, and overall knowledge in students.^4^ Simulation has been widely trusted around the world as a teaching technique to ensure patient safety because it allows a student to practice the skills and apply the knowledge that they have acquired in real-life scenarios.^1^,^4^ Simulation-based teaching facilitates the explanation of intricate concepts and ideas interactively.^25^,^26^ Additionally, incorporating simulation into teaching improves educators’ skills.^27^ A mixed-method study conducted on undergraduate nurses found that the integration of simulation-based learning helps student nurses to be more self-confident, professional, and productive.^28^ Adult learners prefer physical participation in their learning.^28^ Simulation-based learning offers active and participative learning by linking new information with previous knowledge along with immediate feedback from the preceptor, resulting in a deeper understanding of knowledge.^11^ It focuses on repetition, which allows the learner to refine their skills and empowers them to develop skill acquisition, competency, and maintenance. Besides that, it also strengthens the student’s confidence and satisfaction.^11^ The application of simulation in learning demonstrated a positive influence on gaining profound knowledge, self-sufficiency, confidence, and psychomotor skills for the learner.^15^ Problem-based learning through simulation is an outstanding learning process that allows learning with collaboration and engagement among students.^8^,^25^,^26^ Besides the learning advantages, the simulation is an ideal tool to assess students’ competence, such as their professionalism as well as their theoretical and practical knowledge.^29^

### Disadvantages of simulation based education:

Top of FormBesides the numerous advancements and benefits in learning and training, there are also some serious obstacles and drawbacks that are associated with simulation-based learning. No matter how closely a simulation resembles a real patient, it cannot mimic the human body completely;^30^ likewise, various circumstances are challenging to replicate. Hence, there is a possibility of freezing and losing judgments in the actual scenarios when simulated situations become real.^30^ Additionally, poorly designed manikins or simulators, such as lacking the relevant physiological signs and inadequately trained or qualified educators with no specified learning objectives and goals, can have a negative impact on students.^4^ The simulated environment offers approaches to encourage learning, but there is no guarantee that knowledge will be transferred and retained by the students.^30^ The student cannot have real communication with the patient, which will grant them a possibility to eliminate steps like consenting, explaining the procedure, and being empathetic toward patients.^4^ A few more substantial drawbacks include the significantly higher costs associated with high-fidelity simulators, dedicated space for them to operate, and the constant demand for updates and maintenance of both hardware and software simulators which make them unaffordable at many teaching facilities.^30^,^31^ Another consideration that seems to be overlooked is the time factor. Setting and implementing simulations for learning is very time-consuming for an educator and an additional burden on the challenging curriculum of health care.^4^ It is noteworthy to mention here that within current literature, there is an obvious lack of good quality supporting evidence on the outcomes, consequences, and efficacy of simulation-based training.^4^

### Current uses of simulation in healthcare education:

Top of Form Simulation has grown to be a substantial part of the healthcare curriculum and appears to be here to stay. It is a safe learning method equipped with immediate feedback to ensure students achieve skill competency.^15^ A number of articles establish evidence that simulation has already been integrated into several health care programs. The following are a few examples of faculties enhancing learning with the integration of simulation. In emergency medicine, simulation has been determined to be beneficial in academic and professional spheres of learning. Students are trained for critical resuscitation procedures such as emergency airway management, surgical airway management of pneumothorax, and advanced cardiac life support by adopting simulators.^32^ To overcome the anxiety, enhance the confidence, and improve communication, educators instruct mental health students with the assistance of standardized or simulated patients (SPs), also called “trained actors.” It helps students learn how to competently assess a patient’s mental health status, communicate, document findings, and collaborate with the interdisciplinary team to promote optimal patient outcome.^4^ With the advancement in surgical skills, several simulation techniques have been used globally to teach surgical skills, like suturing of banana peels to laparoscopy simulators.^33^ Recently, numerous healthcare professionals have also demonstrated the effectiveness of simulation-based training in managing COVID pandemic.^34^ Simulation programs are also frequently used for anatomy instruction over learning with animal models and cadavers. The concepts of virtual anatomy models are rapidly achieving recognition that empowers students to learn and practice operative skills virtually.^15^

It is apparent and supported by the evidence that the utilization of simulation by faculties is not solely enhancing technical skills but also yields significant improvement in other professional skills, including communication abilities,^33^ the applicability of ethical values and teamwork.^32^

## DISCUSSION

Simulation-based education has become an increasingly popular teaching and training method for healthcare professionals. This review addressed both the benefits and drawbacks of simulation in light of the evidence and how the advantages outweigh the disadvantages, making simulation an appropriate learning tool in health care. The effectiveness of the simulation approach has been evident from the fact that many faculties have broadly integrated simulation into the various academic and clinical curricula. Simulation technology can provide a safe and controlled environment to practice clinical skills, and make decisions with certainty. It is evident that simulation-based training can improve learners’ clinical knowledge and help them acquire skills and knowledge that may not be possible to obtain in traditional clinical settings. The review indicates that simulation-based assessments can also provide learners with immediate feedback, which can help them identify areas for improvement and guide their learning, corroborating the ideas presented by several internationally published studies.^34^

This review found that most research focused on simulation based teaching and not on integrating it into existing teaching methods. To accomplish most of the simulations, educators find creative ways to integrate simulations to create an engaging and immersive learning environment. The purpose of applying these strategies is intended to ensure success for both faculty and students also stipulated by Aebersold and Chang et al.^15^-^39^ One of the recommended strategies is a six-step approach to curriculum development with the purposeful integration of simulation. The fundamental principles of these guidelines are general need assessment (to build the foundation and rationale of your curriculum); target need assessment (polishing the foundation); goals and objectives (concentrating on the purpose of teaching); educational implementation strategies (method and content of the curriculum); and assessment and feedback (to assess the achievement of specified objectives).^35^

The current generation is tech-savvy as they grew up in a world with advancing technology. Virtual Reality (VR) simulation is an innovative simulated teaching strategy. A number of studies have been conducted to justify the use of virtual reality (VR) technology to improve surgical skills and competency in their future role. A virtual world is a “computer-based, simulated multi-media environment”.^12^ A popular and sophisticated example of virtual reality is MUVE (multi-user virtual environments) called “Second Life”. Second Life is an online virtual world based on 3D modelling software that allows residents to build virtual objects and interact with other users simultaneously.^12^

### Debriefing in simulation:

In medical education, debriefing is a potent tool and an effective educational strategy. Debriefing after a simulation-based activity is the most effective way to clarify and strengthen the concepts and lessons acquired in simulation-based education.^36^ It is a cornerstone of the learning experience in the medical setting to improve future training and performance through reflection and feedback.^15^ While the evidence-based gold standard debriefing technique is yet to be determined, some of these techniques include direct observation, debriefing scripts, or video review.^36^ The indication of actively facilitated debriefing is participant engagement completely in the activity to achieve a better understanding of the knowledge acquired in the simulation training and be able to implement it in a clinical setting.^37^ Debriefing is essential for a better outcome in simulation-based learning. Many research studies in the past have provided evidence of a positive learning outcome for healthcare professionals.^36^,^37^

A sound simulation debriefing allows learners to gain a clear understanding of their actions to promote learning outcomes and enhance future clinical performance. Similarly, a poor debriefing session may harm the educator-learner relationship, compromising clinical learning and performance.^38^

### Future research for simulation:

Simulation-based learning research will contribute towards the better training and assessment of healthcare students and professionals. With further investigation and advancement in technology, simulation-based training sessions can facilitate competency-driven clinical training and maintenance of licensure. That will enhance communication skills and encourage students to work inter-professionally, eventually improving patient outcomes.^2^ The number of reviews published in recent years suggests an increased interest in simulation-based learning in health care, but future research needs to emphasize on learning outcomes of simulation from a patient’s perspective;^11^ furthermore, analyzing and comparing the conventional teaching method to the simulation teaching approach.^39^ Currently, despite the recognition of the debriefing process, and considerable research conducted on the effectiveness of debriefing, barely a small proportion of this has reached peer-reviewed journal publication. Future in-depth research needs to clarify the importance of debriefing before and after the simulation-based scenario for bridging gaps and improving communication skills among healthcare professionals to achieve a positive learning environment.^37^

### Limitations:

This literature review has some limitations that should be taken into account when interpreting the results of studies. First, the majority of published studies on healthcare simulation are positive, which may indicate the presence of publication bias. Negative results may be underreported or not published at all, leading to an overestimation of the effectiveness of healthcare simulation. Many studies on healthcare simulation focus on short-term outcomes, which limits the ability to assess the long-term impact of healthcare simulation on clinical practice and patient outcomes. Moreover, most studies on healthcare simulation are conducted in controlled settings, such as academic institutions, which may not reflect the realities of clinical practice in different settings. Further studies are needed on a much larger scale for new teaching and learning methods with practical evidence on the learning outcomes for students to improve and learn new skills and ensure patient safety and compliance.^4^

## CONCLUSION

Simulation-based learning is the definite future of education and is rapidly growing at all levels of health care practice. Although there is a substantial amount of literature available regarding simulation, further research is needed to discover answers to the unknown and reveal additional benefits of simulation as an effective teaching and learning approach to integrate it completely in the health care curriculum.
